# Intelligent Video Analytics for Human Action Recognition: The State of Knowledge

**DOI:** 10.3390/s23094258

**Published:** 2023-04-25

**Authors:** Marek Kulbacki, Jakub Segen, Zenon Chaczko, Jerzy W. Rozenblit, Michał Kulbacki, Ryszard Klempous, Konrad Wojciechowski

**Affiliations:** 1 Polish-Japanese Academy of Information Technology, 02-008 Warsaw, Poland; 2 DIVE IN AI, 53-307 Wroclaw, Poland; 3 School of Electrical and Data Engineering, University of Technology Sydney, Ultimo 2007, Australia; 4 Department of Electrical and Computer Engineering, The University of Arizona, Tucson, AZ 85721, USA; 5 Wrocław University of Science and Technology, 50-370 Wroclaw, Poland

**Keywords:** intelligent video analytics, edge AI, visual transformers, human activity recognition, video surveillance, pose-based HAR, tracking-based HAR, spatio-temporal-based HAR, deep learning-based HAR

## Abstract

The paper presents a comprehensive overview of intelligent video analytics and human action recognition methods. The article provides an overview of the current state of knowledge in the field of human activity recognition, including various techniques such as pose-based, tracking-based, spatio-temporal, and deep learning-based approaches, including visual transformers. We also discuss the challenges and limitations of these techniques and the potential of modern edge AI architectures to enable real-time human action recognition in resource-constrained environments.

## 1. Introduction

Independent security systems, known as security or CCTV cameras, register video footage, and video surveillance cameras monitor specific areas. A single security camera typically produces fifteen to sixty pictures a second, resulting in 3 million images daily. Global information provider IHS Markit reports that in 2015 in the UK alone, 5 million CCTV cameras recorded 25 billion hours of video sequences. Supervisors registered around 350 million operating security cameras worldwide until 2016. In 2017, there were 176 million surveillance cameras in China’s private and public sectors, increasing to 2.7 billion by the end of 2022 [1].

Over the past twenty years, video surveillance systems based on CCTV have become a widely used and effective method of deterring, preventing, and detecting crimes [2,3,4]. Monitoring solutions have migrated from single-unit solutions to intelligent multi-camera network structures, including edge-based architectures of wireless video sensor networks [5] with security and bandwidth constraints [6]. The scope of application and operation of video surveillance systems is extensive, even if limited to human activity recognition. Systems often require complex implementation procedures, resulting in the need to employ specialized surveillance companies to ensure correct security during mass events [7]. Statistical data confirm that the introduction of CCTV systems to monitor areas with an increased level of risk allows for a significant reduction (even by 50%) in the number of robberies and acts of antisocial behaviour by increasing the effectiveness of the services responsible for the implementation of security tasks [8,9,10,11]. Nevertheless, the same reports indicate that the current surveillance systems also possess many imperfections: low credibility of the recorded content, often resulting from systems’ poor technical capabilities or difficult registration conditions, the unreliability of the human factor, and the immaturity of legislative procedures in monitoring and response. The essence of monitoring concerning the security aspect of citizens should be an efficient flow of complete information between system operators and the police [12,13].

The number of video surveillance system installations and the amount of information collected is rapidly increasing, creating problems in collecting and processing information by supervisors. Research shows that, on average, after twenty minutes of observing one screen, the operator may overlook 90% of what is happening in the monitored place [8]. The current development directions of IP CCTV solutions [5] are systems of intelligent analysis of dynamic scenes with the automatic detection of many moving objects [14] and understanding their behaviours [15,16,17]. Due to the functional requirements, the market distinguishes the development of active and passive video surveillance systems. Passive systems usually record the monitored zone’s video stream for evidence in the event of a crime. Active systems support the supervisors with additional information from the presented or processed image. The most advanced research concerns IVA [18]. Such systems attempt to obtain a description of events in the monitored zone and then take appropriate actions based on the interpretation of monitored events [16]. The necessary image registration and processing are associated with difficulties analogous to those occurring in computer vision systems, remarkably the variability of illumination, observation point, orientation, and distance from the observed object. It is challenging to build a general-purpose intelligent surveillance system [19] and continuously supply it with electrical power [20]. That is why professionals adapt existing systems to specific requirements [21,22]. The most significant difficulty is developing generalized algorithms to solve specific IVA-related problems. Therefore, intelligent surveillance systems usually comprise a library of modules with algorithms for specific applications [23]. The increasingly popular no-code/low-code computer vision platforms reduce the entry threshold into computer vision-based solutions for non-professionals, where applications are built visually from developed components. Gartner predicts [24] that by 2024 more than 65% of applications will be developed with no/low code development.

This survey provides a comprehensive overview of HAR methods chosen specifically for potential use with surveillance cameras, categorizing them into four distinct groups. It also discusses the advantages and disadvantages of each group, including their efficiency and suitability for IVA applications. Finally, it synthesizes the most recent and relevant research on these methods, providing readers with up-to-date insights into the strengths and limitations of each class of methods. The paper offers information on suitable datasets to make models more useful for practical use in intelligent video surveillance challenges. This will make it easier for readers to comprehend the value of data in creating HAR models and allow them to choose relevant datasets for their unique IVA applications. It is a helpful resource for academics and industry professionals who want to enhance the efficiency and dependability of IVA systems for the challenge of human action recognition techniques from the monocular camera in video surveillance.

## 2. Intelligent Video Analytics

The typical workflow in intelligent video surveillance systems includes the following stages: image acquisition, object and motion detection, object classification, object tracking, analysis and understanding of behaviour and activity, people identification, and information fusion in multi-camera systems [25,26,27,28,29,30,31].

Virtually every intelligent surveillance system detects objects and motion. Motion detection requires segmenting adjacent areas representing moving and stationary objects [32]. The most popular approaches to motion segmentation include temporal differencing, background subtraction, and optical flow [25,26,31]. Statistical background subtraction is a more frequently used segmentation method due to its resistance to disturbances, shadows, and changes in lighting [33,34]. Researchers usually use optical flow [35] to detect the movement of a point or specific feature [36] in a video sequence using traditional or modern methods such as FlowFormer [37] or PanoFlow [38]. However, most optical flow approaches are unsuitable for real-time applications because of their vulnerability to interference and difficulty in putting algorithms into practice. The unequivocal detection of moving areas in a video sequence allows attention to focus on these areas during subsequent processes, such as tracking or behaviour analysis, and speed up the entire processing process [39,40]. The subsequent processing steps, including object tracking, behaviour analysis, and recognition, strongly depend on the detection effect.

An unambiguous classification of moving objects is necessary to track them and analyse their behaviour accurately. Classification is understood here as a standard pattern recognition task. The most popular categories of approaches used to classify objects include classification based on recognized shape and motion [26]. Motion classification is sometimes greatly facilitated because, in general, human movement exhibits periodic properties.

Table 1 provides a broader context for using HAR methods in IVA systems by outlining the different workflow elements and operations involved in implementing such solutions. As one can see, the HAR particular classes of recognition strategies described in the paper are just one part of the overall workflow, which includes data acquisition, pre-processing, object detection, object tracking, event detection, decision making, and alert generation. Depending on the specific use case, some or all of these elements may be present in an IVA system. Tracking an object in a system with many cameras in real-time under changing conditions is a complicated task [28]. The object tracking task uses the classification results. It becomes more complex when more moving objects [15,41,42,43] appear on the scene, which is treated as a background when tracking the selected object [44]. We can treat the tracking problem as a correspondence problem finding a visual object in two consecutive image frames [45]. The position of an object during tracking is usually transformed into 3D coordinates. We can divide tracking methods into four main categories based on [26,44,45]: regions, contours [46], features [47], model, and a hybrid that combines the advantages of region- and feature-based methods or a combination of these methods. Sequential Monte Carlo methods [48], particularly condensation algorithms [49], dominate the group of generalized tracking methods.

Understanding and interpreting movement plays an essential role in intelligent surveillance systems. Recognition of human movement from the video stream starts the process of extracting information about movement from an image sequence. The surveillance system can learn patterns of movement, e.g., walking, extracting the features that determine movement dynamics by decomposition of a tracked movement [50,51,52,53]. There are three leading methods of extracting motion information from an image sequence: information from optical flow feature analysis [54], information from trajectory-based features [44,55,56,57], and information from region-based features [58].

The CCD, thermal imaging, and night vision cameras are the three most popular image recording devices in surveillance systems [19]. Simultaneous acquisition and presentation of images from cameras of various technologies, such as vision and thermal imaging, ensure optimal day/night vision in various weather conditions [59]. The separate processing of image information results in individual and different results with the inherent flaws of each image acquisition technology. In the case of vision cameras, these provide low-contrast images at night, in bad weather, and at long distances. In the case of thermal imaging cameras, low resolution, poor contrast in rainfall as well as ambiguity in the intuitive interpretation of the images from long distances. Data fusion, i.e., the superimposition of images from cameras of different technologies and the presentation of the resulting image on one screen, improves image quality, eliminates the weaknesses of the combined technologies, and increases the efficiency and comfort of the operator [59,60]. In surveillance systems, there is also a need to simultaneously present the image from many cameras partially covering the viewing areas or automatically switch such cameras when the tracked object appears in the field of view of another camera. Using photogrammetric measurement methods from multiple cameras at the initial image processing stage facilitates the exact data fusion process [61,62].

The assessment of motion detection performance, object tracking [33,63], object classification, intent detection, behaviour, and identification in intelligent video surveillance systems are complex, but the performance impacts the product. The performance is one of the leading topics of the annual challenges around PETS [22], or currently ActivityNet [64] and MMAct [65], with many algorithms, strategies, and benchmark datasets. The 2D PETS datasets include indoor and outdoor human and vehicle tracking with single and multi-camera, posture classification, facial expression, and interaction. ActivityNet is a large-scale activity recognition challenge that aims to cover many complex human activities in people’s daily lives. MMAct is a multimodal dataset for action understanding based on diverse modalities.

Companies’ current area of interest concerns developing methods and analyses that effectively detect human and object behaviour based on activity patterns [66,67]. Most current video surveillance systems use solutions that allow for effective image processing, mainly in motion or object detection and object tracking in single-camera systems. Object tracking works well in open terrain, but its effectiveness drops when more than one object is in the scene or occlusions occur [68]. The manufacturers of current systems focus mainly on defined patterns of behaviour. This approach shows low effectiveness and often leads to many ambiguities in the events identified by the system. Essential strategies for developing intelligent video surveillance components in the next decade will include interpreting tracked objects in 3D space and advanced real-time behaviour analysis by the adaptive discovery of behaviour patterns.

In recent years, edge processing [69] has grown in popularity, and many large companies have developed small chips to suit the image processing workload. The most well-known products are Google Coral™, Intel Movidius™, NVIDIA Jetson™, Qualcomm Snapdragon™, Apple A-series™, Xilinx Alveo™, and Kneron™. A separate group of deep learning solutions is lightweight image recognition algorithms and the related edge AI trend. The principle of operation of each of the edge processing products is similar. The software includes a dedicated optimizer model that takes models pre-trained in the MXNet [70], TensorFlow [71], Caffe [72], ONNX or other less popular frameworks. The available models can recognize images, human faces, bodies, or objects, on the edge device integrated with the computer system. The software transforms them into a simplified internal representation of a specific architecture. These are hardware architectures for the aforementioned four products: tensor processing unit, vision processing unit, graphics processing unit, and neural processing unit. The particular inference engine loads the simplified representation of the model. The efficiency of inference is so low that it is not yet suitable for solving human action recognition problems in real-time from a video stream of at least at 25/30 fps. However, one of the disadvantages of edge AI recording and analysis is the cost of cameras with sufficient computing power. At the same time, having cheaper cameras and leaving the processing to the server can be less costly.

Many surveillance architectures evolved from the cloud to the edge model and are called hybrids. Edge cloud design [73] is increasingly seen as a natural advancement of cloud computing and an enabler of the coming industrial revolution with the widespread IoT. It includes fog computing, cloudlet, mobile edge, micro data centres, and many others [74]. Video analytics over the cloud offers the benefits of server systems, such as centralized, top-down control, and advanced AI analytics, but without server costs and maintenance needs. It is usually provided as video surveillance as a service (VSaaS) model. Hence, there is no upfront cost, including video recording, storage, remote viewing, management alerts, and cybersecurity. The main differences between the most popular IVA architectures are present in Table 2.

The rest of the paper describes different HAR methods that can be used as an algorithmic component of an IVA system. The methods are grouped into four categories: pose-based methods, tracking-based methods, spatio-temporal-based methods, and deep learning-based methods. For each group of methods, the paper discusses the pros and cons of using them for HAR, including their accuracy, efficiency, and suitability for different applications. Overall, the paper provides a comprehensive overview of different HAR methods that can be used in an IVA system and provides valuable insights into each method’s strengths and weaknesses, helping readers make informed decisions about which method may be best suited for their particular application.

## 3. Human Action Recognition Methods

The topic of HAR in videos is an increasingly popular field, as evidenced by the number of publications each month. Google Scholar collated 10,000 scientific articles published between 2020 and 2022. on HAR. More detailed information on HAR provides extensive literature with various methods and comprehensive reviews [75,76,77,78,79,80,81,82,83]. The following summary presents only the selected aspects and main directions in this area to determine the selection of research directions needed to develop the methods and algorithms necessary to choose the method more effectively.

Workflows of various approaches for HAR can differ significantly, especially regarding the methods used for feature extraction, action segmentation, and classification (Table 3).

Pose-based methods typically extract features related to joint positions, angles, and velocities and may involve techniques such as skeletonization and joint detection. Action segmentation may include detecting the start and end of action sequences based on changes in common positions or velocities.

Tracking-based methods may use motion-based features such as velocity, acceleration, and trajectory and may involve techniques such as optical flow or motion history images. Action segmentation may involve detecting changes in motion patterns or transitions between different motion patterns.

Spatio-temporal-based methods typically involve extracting features that combine motion and spatial information, such as optical flow or histogram of oriented gradients (HOG). Action segmentation may involve detecting changes in movement and spatial patterns or transitions between different spatial and motion patterns.

Deep learning-based methods typically involve using deep neural networks to learn features from raw image or video data automatically. They may include techniques such as convolutional neural networks (CNNs), recurrent neural networks (RNNs), or transformers. Action segmentation may involve using RNNs to learn temporal patterns in the data and segment action sequences. Thus, while there may be some overlap in the workflows of different HAR approaches, the specific methods and techniques used can differ significantly, depending on the system.

Transfer learning [84] and continual learning [85] are essential concepts in deep learning-based methods for HAR. Transfer learning involves using pre-trained models to transfer knowledge to new tasks. In contrast, continual learning, also known as lifelong learning [86], consists of updating a model incrementally as new data becomes available. Other related concepts include federated learning [87], which involves training a model across decentralized devices, and gossip learning [88] federated variant that does not require an aggregation server; multi-task learning, which consists of preparing a single model to perform multiple associated tasks; ensemble learning [89], which combines various models to make predictions; and reinforcement learning, which involves an agent learning to make decisions in an environment through trial-and-error interactions. Researchers are exploring ways to incorporate these concepts into HAR algorithms to improve performance and efficiency.

The field of visual analysis of human actions and behaviour [90,91] currently has broad practical applications in industry, medicine, and surveillance. The biggest market includes IVA [68,92,93,94], but also monitoring and supporting systems for ambient-assisted living [95,96,97,98] and rapidly growing applications involving visually controlled interactions between people and robots [99,100,101].

The dynamic development of the evidence management market [102] has increased cameras to over 1.5 million worldwide. According to IDC’s predictions [103], the global amount of data created, captured, and replicated worldwide will increase to 175 zettabytes (1 zettabyte = 1 trillion gigabytes) by 2025. There are still many open problems where the task of human action recognition is far from being solved.

### 3.1. Pose-Based Methods

Detecting, associating, and tracking human skeleton keypoints is a computer vision problem involving human pose estimation [104] and tracking. Significant processing resources required to execute skeleton keypoints tracking in live video data limit the precision of human posture estimation results in real-time. Thanks to recent advancements, new real-time applications are now conceivable. As a result, state-of-the-art approaches often rely on customizing CNN architecture for human posture inference. As depicted in Table 4, the workflow of common elements and operations for human action recognition using pose-based methods consists of several stages, including pose estimation, feature extraction, and classification.

The two most popular strategies for pose-based methods are top-down and bottom-up. The top-down approach begins with a person detector and estimates body parts inside the identified bounding boxes. The bottom-up approach begins by estimating each body part individually, then grouping them to create a unique configuration. Among models suitable for pose-based methods, three are the most popular [105]: Kinematic or skeleton-based for 2D and 3D pose estimation, volumetric for 3D pose estimation, and planar or contour-based composed of one shape or geometric body parts. Recent research has also addressed reliable tracking and pose estimation in natural scenes. Table 5 shows a comparison of several pose estimation methods based on their accuracy, the number of joints they can estimate, their approach (e.g., top-down or bottom-up), and their backbone architecture. The table provides a useful overview of the strengths and weaknesses of each method. The most established 2D real-time multi-person keypoint detection is OpenPose [106], and its faster commercial competitor wrnchAI. Next, are the AlphaPose framework [107,108] and Mask R-CNN [109] based on feature pyramid network (FPN) [110] and a ResNet101 backbone [111]. HRNet [112] maintains high-resolution representation for pose estimation, while DeepCut [113] follows a bottom-up approach, detects people, and subsequently estimates their body configurations. DeepPose captures all joints and uses deep neural network regressors for pose estimation [114], and DensePose maps all human pixels from RGB image to its 3D body surface [115].

BeomJun et al. [124] compared and analysed the major pose estimation frameworks. Pose-based methods for HAR use an explicit skeletal representation for motion description. The topology of the human skeleton is an important parameter. YOLOv7 [118], a one-shot multi-person pose detector, has a topology with 17 landmarks for a single person, while MediaPipe [116] has 32 keypoints for a single-person skeleton. These methods estimate human pose by identifying skeleton anatomical joints or keypoints in each video frame. Video frames have a sequential nature, so using RNNs, such as Bayesian CG-LSTM [125], hierarchical bi-RNN [126] or AGC-LSTM [123] and graph convolutional networks [122], has made these architectures very common. We do not recommend using skeleton-based representations to describe human-like objects moving in a real-life environment with constraints causing regular silhouette occlusions. We mentioned important works because pose-based methods represent one of the main promising directions for HAR. More information and many open problems connected with this direction are addressed in the following surveys [105,127,128,129,130,131].

### 3.2. Tracking-Based Methods

While developing an effective system for tracking humans in the video stream, one should address some considerations. The tracking-based methods [132] must be capable of following the tracked human object even under visually difficult situations such as changing illuminations, occlusions, cluttered backgrounds, and complicated human movements, all of which can cause tracking issues. In addition to changes in the environment where a human object is found, the human object can change itself. Such change calls for a consistent tracking system to possess a mechanism that can adapt to the actual human object’s appearance. Table 6 provides an overview of the workflow, common elements, and operations used for human action recognition with tracking-based methods.

A system dealing with live video must be able to handle data quickly. The speed of the viewed object determines the processing speed, but at least 25 fps must be established to provide a near-real-time effect. As a result, a quick and efficient implementation is essential, as is the selection of high-performance algorithms. Tracking algorithms can use visual features [133] such as histogram of gradient (HOG) [134] colour [135], Haar [136] and popular learning methods such as support vector machine (SVM) [137], or ensemble learning methods, e.g., boosting [138]. To localize objects, deterministic methods [135] and stochastic methods [138] have been used. Compensation for appearance changes can be achieved using robust mixture models [139] or online boosting [136]. An additional problem is the minimization of the occlusion of surrounding objects [140]. Current reviews of classic object-tracking methods are included in [141,142]. Most of the selected object-tracking methods refer to two categories:

1. Tracking methods using detection (e.g., [143]). For the assignment methods, optimal allocation methods, such as the Hungarian, optimal flow, or graph-based discrete optimization methods, are used. These methods recognize each tracked object in each frame and then group objects from consecutive frames so that each group creates a separate trajectory. The group of tracking methods using detection include:
  (a) a multiphase cascade method with a moving time window [144];
  (b) methods based on a generalized solution of optimal cliques in generalized minimum clique problem (GMCP) graphs [145] and globally optimal generalized maximum multi clique (GMMCP) problems [146];
  (c) a method of generalized linear allocation of short GLA tracklets [147];
  (d) methods based on the estimation of the similarity measure of ADMM dynamics [148] i IHTLS [149];
  (e) a simultaneous tracking method with object segmentation, using a multi-label conditional random field to determine the optimal set of trajectories [150];
  (f) a method to detect and track homogeneous objects with high density [151] using gradients and contours;
  (g) a machine learning method with appearance discrimination using high-certainty tracklets [152], and incremental linear discriminant analysis [153].
2. Methods of tracking using correlations whose main advantage is the speed obtained by using FFT:
  (a) a robust tracking method with accurate scale estimation [154] using a HOG descriptor [134]. The method uses a discriminative correlation filter in the MOSSE method [155]. The method [154] is faster than competitive methods: 2.5 times faster than [156], 25 times faster than ALSA [157], and 250 times faster than SCM [158].
  (b) The method [159] uses dense space-time context learning for tracking.
  (c) The fast-tracking method with kernel correlation filters [160] based on HOG descriptors.

An alternative tracking approach is represented by methods based on clustering trajectories:

1. the method [161] clusters paths constructed from points detected by the SURF detector using the SIFT/SURF descriptor for comparison;
2. the method [42] clusters paths constructed from points of extreme contour curvature.

A typical single- and multi-object tracking approach uses a detector for object localization and re-identification for object association. Hundreds of methods have been competing in SOT [162], GOT [163], MOT, and MOTS challenges since 2015 [164,165,166,167,168]. Recent trends indicate interesting directions into trackers derived from deep learning-based transformers [169,170,171] applying visual attention [172] for object tracking. Some authors address similar methods for long-term tracking scenarios [173,174,175]. For the comprehensive, up-to-date summary, this [176] work investigates the present state of DL-based visual tracking algorithms, evaluation metrics, and benchmarks in-depth with leading visual tracking methods.

### 3.3. Spatio-Temporal Methods

This section provides an overview of the selected and most representative space-time methods for action recognition in temporally and spatially trimmed videos. Algorithms and methods have been improving over the years, and the problem shifted from recognizing actions in videos recorded in laboratories to realistic datasets such as HMDB51 [177], UCF101 [178], Hollywood2 [179] or VMASS [180], MCAD [181], surveillance camera fight [182], and RWF-2000 [183] datasets created directly from surveillance cameras (Section 3.5).

**Figure 1:**
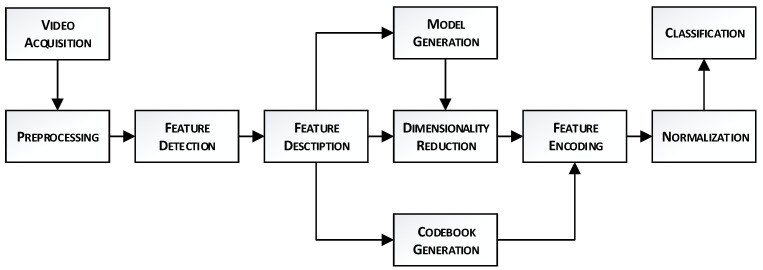
An illustration of the bag of visual words approach pipeline.

Table 7 shows that deep learning methods can be considered spatio-temporal-based approaches too. There are certainly many other deep learning methods beyond those described in the above table that could be used to analyse human movement. However, the focus of the table is to provide an overview of some commonly used and effective deep learning methods for HAR rather than to provide an exhaustive list. After conducting broad research with many different spatio-temporal-based HAR methods [184], we focus in this section on the bag of visual words (BoVW) approach to presentation among the most promising and actual classic spatio-temporal methods. The BoVW approach has been widely used and benchmarked for human action recognition and is still relevant today. By describing BoVW in detail in the spatio-temporal-based section of the table, we highlight the fact that it is one of the foundational approaches in this field and has been used as a benchmark to compare the performance of newer deep learning methods. For BoVW in a video stream, a part of an image is the visual equivalent of a word, and it can be represented by a bag of quantized invariant local descriptors [185]. This approach provides a flexible choice of processing algorithms using features computed independently over each set of automatically detected RoIs. Finally, such a method structure is easily scalable and robust to the occlusion of motion regions, representing people or partial visibility in a video stream. Figure 1 presents the BoVW approach pipeline divided into ten steps. First, the videos are acquired and annotated for a supervised learning problem.

Pre-processing is optional. The videos can always be unified, e.g., rescaled or compressed. The feature detector chooses areas in the video that are volumes for computing features. Among the most popular are DI [186] and STIP [187], which provide sparse representation. However, using feature detectors is optional. Random sampling [188] and dense sampling [189] do not detect regions for feature extraction, which speeds up the final method. According to the current research, the dense sampling approach outperforms STIP [189]. Next, features within these sub-volumes are computed by the feature descriptor, popular descriptors include HOF, HOG [190], MBH [191], and fast GBH [188]. In the next phase, the dimensionality of the features is reduced by the popular PCA algorithm, which is a crucial element for performance [192]. Feature encoding clusters similar descriptors. Here, simple *k*-means and BoVW histogram or GMM and FV are utilized. These methods need a model and codebook to be established. The PCA model and codebook are usually learned from a subset of descriptors, e.g., as in [193]. The final representations are normalized and classified, usually by SVM with the RBF-$\chi^{2}$ kernel for BoVW histogram descriptor and linear for FV. Many methods utilize different algorithms and combinations for each described phase of this approach. Some selected methods are presented below. This description is informative to analyse the method flowcharts in Appendix A. Unless stated otherwise, the methods mentioned use GMM with k = 256, FV, and SVM. The authors of the above cited examples often provide a study of different parameters. We take into account only the best reported results.

**Figure A1:**
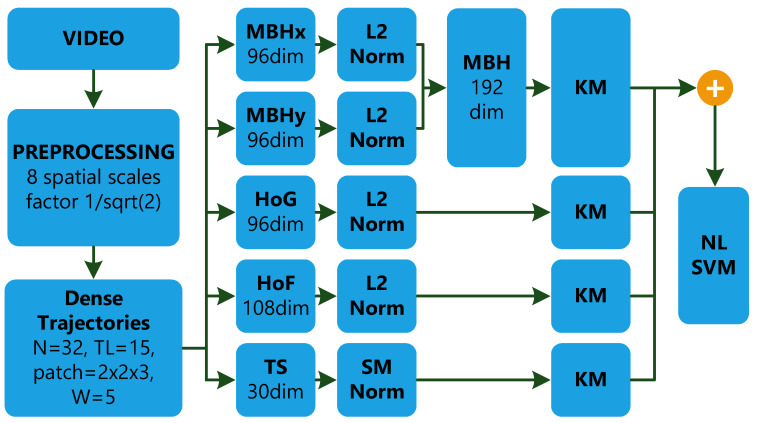
An illustration of the dense trajectories method [194]. TS: Trajectory shapes; SM Norm: Norm by Sum of Magnitudes; KM: *K*-means with *K* = 4000, 100,000 randomly selected features performed eight times, selected result with the lowest error; NL SVM: Non-linear SVM with the RBF-$\chi^{2}$ kernel.

**Figure A2:**
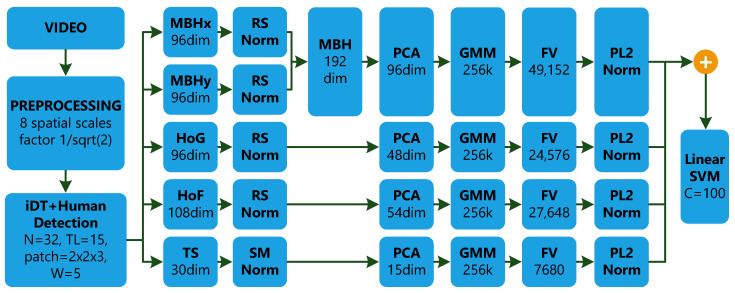
This figure illustrates the backbone for two different approaches to HAR. Variant 1 uses the improved dense trajectories method [195], which includes Trajectory Shapes (TS), Norm by Sum of Magnitudes (SM Norm), Gaussian Mixture Model (GMM), Root SIFT Norm (RS Norm), Fisher Vector (FV), and Power and L2 Norm (PL2 Norm). Variant 2 is an extended method that builds on Variant 1 and incorporates multi-skip feature stacking [197]. It uses iDT + Human Detection to extract descriptors and skips frames to reduce the number of frames processed. Skipping each 2nd frame adds 0.5 more frames and descriptors, equivalent to having a 0.5 longer video, while skipping each 3rd frame adds 0.3 more frames, and skipping each 4th frame adds 0.25 more frames. Variant 2 includes RS Norm, TS, SM Norm, GMM, FV, and PL2 Norm.

**Figure A3:**
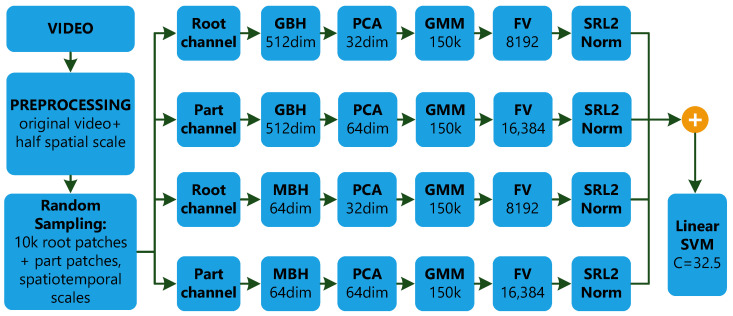
An illustration of the gradient boundary histogram method [188]. Random Sampling: A total of 10,000 root patches at half spatial resolution, eight (2 × 2 × 2) overlapping part patches. Eight spatial and two temporal scales. Initial patch size 28 × 28 × 14. Each patch is subdivided into 2 × 2 × 2 cells, which together with eight bins gives 64 dim descriptor TS: Trajectory shapes; SM Norm: Norm by Sum of magnitudes; GMM: Gaussian Mixture Model; FV: Fisher Vector; SRL2 Norm: Square Rooting and L2 norm.

**Figure A4:**
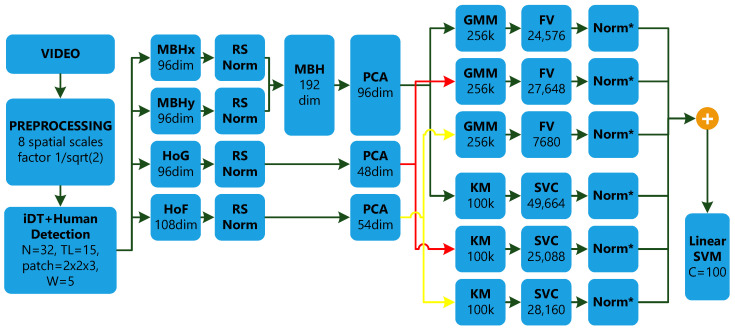
An illustration of the hybrid Peng’s system [192]. RS Norm: Root SIFT norm; SM Norm: Norm by Sum of Magnitudes; GMM: Gaussian Mixture Model; FV: Fisher Vector; Norm*: Power Norm with factor α = 0.5 which is signed square rooting and then intra L2 Norm.

**Figure A5:**
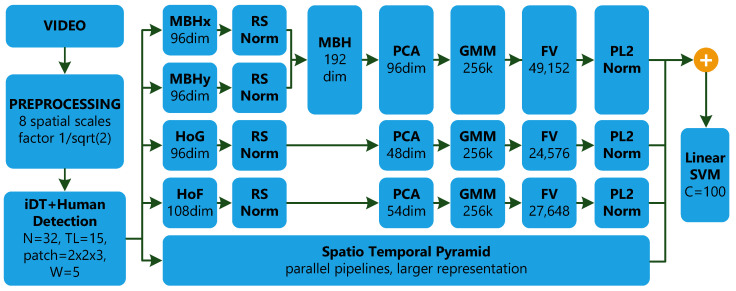
An illustration of the robust and efficient INRIA method [196]. Additional annotations with video, bounding boxes with humans, bring improvement. RS Norm: Root SIFT Norm; Spatio-Temporal Pyramid: Parallel the same steps in smaller cuboids. H3 enlarges the dimension four times, T2 enlarges it three times T2 + H3 enlarges the dimension six times; GMM: Gaussian Mixture Model; FV: Fisher Vector; PL2 Norm: Power Norm with factor α = 0.5 which is signed Square Rooting and then L2 Norm.

**Figure A6:**
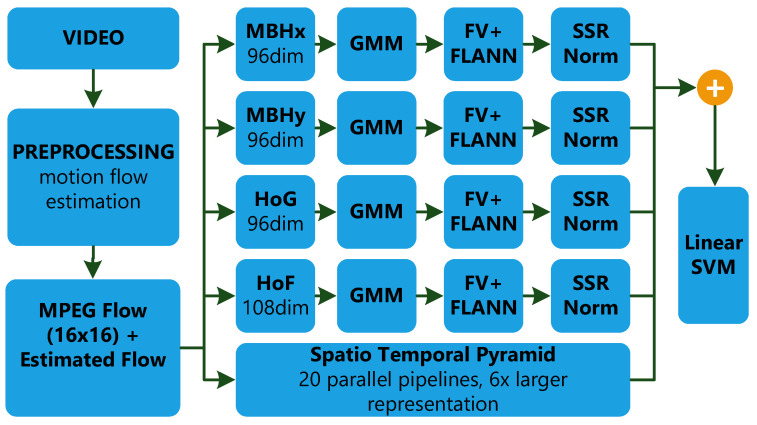
An illustration of the efficient feature extraction, encoding, and classification method for AR [198]. MPEG compression and decompression for estimation of motion flow (used instead of optical flow) during pre-processing step. MPEG flow (16 × 16) + estimated flow (to increase the spatial resolution by a factor of 2). A very comprehensive spatio-temporal pyramid; a total of 24 cells, two scales: 32 × 32 × 15 with spatial stride 16 and temporal stride 548 × 48 × 15 with spatial stride 24 and temporal stride 5 descriptor’s area: 2 × 2 × 3; GMM: Gaussian Mixture Model; FV: Fisher Vector; FLANN: Fast Library for Approximate Nearest Neighbors SSR Norm: Signed Square Root (power normalization with an α factor of 0.5) and L2 Norm.

Heng Wang et al., in their work [189], compared the most popular descriptors, such as Cuboids, ESURF, HOF, HOG/HOF, HOG, and HOG3D, in combination with different detectors such as Harris3D, Cuboids, Hessian and Dense. The authors presented results on the following datasets: KTH, UCF, and Hollywood2. One of the essential conclusions for further research is that dense sampling detectors outperform sparse approaches. Based on the assumption of dense detectors, the dense trajectories method is a source approach for the best up-to-date methods that utilize hand-crafted features. For the most promising methods, flowcharts with the most critical blocks related to Figure 1 have been drawn and shown in Appendix A for visual comparison of the structures. First, the flowcharts for HAR by dense trajectories [194] and HAR with improved trajectories [195] are presented in Figure A1 and Figure A2. The one-against-rest approach is used in these multi-class classification cases, and the classes with the highest score are selected. The gradient boundary histograms for action recognition [188] pipeline in Figure A3 takes advantage of the random sampling method encoding local and the root channel separately. Pengs et.al, in their comprehensive study of BoVW methods [192], proposed the pipeline (Figure A4) composed of different BoVW methods and many different low-level descriptors. An efficient video representation and a robust approach for action recognition [196] combined iDT with spatial pyramid and spatial FV to preserve spatio-temporal features in the video presented in Figure A5. Beyond Gaussian pyramid: Multi-skip feature stacking for action recognition [197] (Figure A2) proposes efficient feature extraction at different time scales, encoding, and classification for action recognition similar to [198] (Figure A6). An efficient and effective human action recognition in the video through motion boundary description with a compact set of trajectories was presented in [199]. The method goes further with improved dense trajectories leading to better accuracy. The motion vector is interpolated between skipped frames to avoid computing optical flow and speed up the method. The following modification is that the number of trajectories per frame decreases below a threshold. A trajectory is also discarded in the case of too little motion within it. Fisher linear discriminant analysis (FLDA) is utilized for further dimensional reduction, working with sparse representation-based classification. Evaluation results demonstrate that there are fewer trajectories per frame than in iDT, and the methods are fast. Accuracy was also improved, but which change was crucial for this result was not evaluated. Action recognition with stacked fisher vectors [200] shows that SFV is effective in combination with standard FV. Here, the iDT is taken as the input, and a two-stage clustering structure is provided. This is a kind of mid-level approach without learning discriminative action parts. At the very beginning, a 396-dimensional descriptor is computed. It combines HOF, HOG, and MBH descriptors in sampled sub-cuboids. There are 600 to 6000 subcuboids, which differ across the datasets. Next, PCA and whitening reduce the dimension to 200. For each sub-cuboid, a consecutive FV is computed. This representation is, in turn, reduced by max-margin and further again by PCA and whitening, having 200 elements in the end. Another FV encodes the descriptors of a cuboid. Single-stage FV and SFVs are complementary. Its combination is one of the best available methods. Uijlings et al. [201] described popular descriptors in more detail and explained how to efficiently implement these algorithms to find a balance between accuracy and speed. The paper’s authors [202] enriched video representation by focusing on encoding objects for actions and obtaining the best result by fusing FVs and SFVs and object-based proposed representation.

### 3.4. Deep Learning Methods

In modern DL architectures used for HAR, information concerning objects is usually included in video frames for the spatial and temporal dimensions of their movement. As shown in Table 8, the workflow for human action recognition using deep learning-based methods includes common elements and operations.

Since 2014, the most popular supervised DL model is the CNN, effectively applied for video HAR when Karpathy et al. [203] proposed a single-stream CNN to fuse temporal information from consecutive frames using pre-trained 2D convolutions. Later, Simonyan and Zisserman [204] presented a two-stream network architecture more suitable for the HAR task. The Simonyan method distinguishes temporal and spatial information using two separate streams for a CNN with three fully connected and five convolutional layers. The spatial part is trained on still images from the ImageNet challenge dataset [205]. The temporal part needs the stacking multiple-frame optical flow to be computed beforehand. The multi-task learning was performed on the most popular benchmarks UCF101 [178] and HMDB51 [177] datasets for the temporal part, and the accuracy of the computed soft-max scores were fused by linear SVM. These two papers form the backbone of most DL methods for HAR, differing in how spatio-temporal information is combined. Many other papers on single-, two- [206] or three-stream [207,208] architectures have evolved from these propositions. The most popular DL-based architectures applicable in HAR are presented in [209]. Due to their high computational complexity, multi-stream architectures are unsuitable for real-time surveillance applications where operators can adjust the system to new recognition classes. DL methods usually need a lot of computational time and data. They are challenging to analyse in detail, but selected variants with working code can compete with other methods in terms of accuracy. Some of the methods extend input from 2D performing 3D convolutions by 3D CNN with spatio-temporal information [210,211]. Combined methods use deep learning and hand-crafted features. Wang et al. in [212] combined the iDT approach with DL features. The best results were obtained by fusing DL descriptor with the traditional iDT approach at the FV level. A similar combination with improved FV (iFV) is presented in [213]. The VLAD [214] was used to encode spatio-temporal descriptors in combination with CNN [215,216,217,218,219]. Optical flow is a useful but inefficient motion model for CNN-based propositions, including two-stream [220,221] or faster modifications [222] and dynamic versions [223]. Some methods use CNN with skeleton sequences [224] to encode spatio-temporal information into texture patterns, others [225] use RGB-D representation for action scene flow. Temporal long-term relations are learned using sequential RNN [226] and LSTM [227,228,229] architectures. The workflow of common elements for human action recognition using deep learning-based methods, including data preparation, network design, and training, is presented in Table 8.

The idea of attention mechanism applied to computer vision [230] tries to estimate dependencies between relevant elements in consecutive video frames according to certain domains trying to learn the most important features or regions in an image or video by assigning different levels of attention to different parts of the input. According to [230], the channel attention mechanism (C) determines the importance of different channels (what to pay attention to), such as colour channels, in an RGB image. Spatial attention (H and W) determines the essential regions within an image based on their spatial location (where to pay attention). In contrast, temporal attention (T) is used to determine the critical frames in a video (when to pay attention). Branch attention combines these different attention mechanisms and provides a more comprehensive attention model.

These attention mechanisms are effective in various computer vision tasks, such as object detection, semantic segmentation, and video classification. By focusing on the most important features, attention mechanisms can help to improve the performance and efficiency of deep learning models in these tasks. Long et al. [231] applied attention to better capture temporal patterns in videos, and Dai et al. [232] proposed a spatio-temporal attention mechanism for feature learning processing to enhance the HAR performance.

The latest DL trend visual transformers (ViT) [233] could be a gamechanger in trying to parallelize operations by replacing the known drawbacks of sequential RNN architectures and, at the same time, limit the bias of locality from CNNs by using self-attention and two-stage training mechanisms. The main elements of ViT are presented in [234]. ViT’s self-attention layer allows incorporating of global information throughout the entire image. To recreate the visual structure from the training data, ViT learns to encode the relative placement of the patches. Transformers lack prior knowledge of visual structure, resulting in increased training periods and the need for enormous datasets for model training. ViT separates the picture into visual tokens, whereas CNN employs pixel arrays. The video transformer network [235,236] for temporal relationships uses a long-former [237] to process the whole video in one pass. Action transformer networks try to aggregate spatio-temporal context cues around a selected person only using RGB frames [235]. Other propositions optimize the method of capturing spatio-temporal relations in videos [238,239]. Plizzari et al. [239] proposed a spatial self-attention module and temporal self-attention transformer for inter-frame correlations to model the human skeleton structure.

Despite the exceptional performance of transformer models for standard HAR benchmarks and intriguing prominent features, there are significant problems related to their practical use. The demand for enormous volumes of training data and the highest computing costs are the most significant barriers. Visualizing and interpreting transformer models has also proven challenging. We present a summary of these problems in this part, along with some recent initiatives to overcome these constraints.

Transformers provide an easy way to see what they are paying attention to [240], while this does not give a complete indication of the types of associations learned by the model [241], it does provide some insight into what it considers significant for specific samples [242]. Few studies have attempted to interpret transformers further than this for vision [243]. We only identified a small portion of research depicting these attention activations for individual samples in the ViT literature.

### 3.5. Datasets for Method Evaluation

There are always complex problems to solve in videos from a surveillance camera, such as changing light conditions, background clutter, and occlusions. Numerous datasets are available for benchmarking and comparing human action recognition methods [244]. The most up-to-date paper [245], published in May of 2022, presents an excellent vast summary with a catalog of the 704 existing multimodal human movement datasets available for researchers prepared in labs and the real world. Table 9 indicates the most popular datasets from real-life scenarios considered when selecting the most representative datasets to evaluate the most promising methods. The state-of-the-art methods often use HMDB51, Hollywood2, and UCF101 for benchmarking. We have followed this direction and extended this set of benchmarks with one additional test with the VMASS2 dataset, where all video streams come from surveillance camera networks in the metropolitan area. Several publicly available datasets have also been widely used in the research community to evaluate the performance of IVA algorithms. Some of the most popular datasets: RWF-2000, XD-Violence, and UCF-Crime, include videos with registered violence. Weizmann and KTH are datasets of human actions and objects captured using a static camera. Weizmann was performed by nine people and KTH by 25 actors. The VMASS dataset includes a diverse range of human actions captured from various surveillance camera angles under different lighting conditions, making it a challenging and comprehensive dataset to evaluate the performance of IVA algorithms. On the other hand, the UCF sport action and UCF11 datasets consist of human actions captured from YouTube videos. The UCF101 dataset includes 101 different human activities, while the HMDB51 dataset contains 51. The key features of the VMASS dataset include its large scale, diverse action categories, and multimodal annotations, which provide a rich resource for developing and evaluating new IVA algorithms. Each of these datasets include a diverse range of human actions recorded using various cameras and under different conditions, making them well-suited for assessing the robustness and accuracy of IVA algorithms.

## 4. Discussion

In this paper, we briefly presented the state of knowledge of modern IVA architectures, which has become a generally available trend in video surveillance systems in recent years.

### 4.1. IVA Systems

Despite the shortcomings of advanced IVA technologies, most systems struggle with the problems of business continuity, efficient alerting and response, and the inability to dynamically track a detected event in the camera network. Despite technological advancements, the current systems do not have modules to effectively recognize the actions and behaviours of a broad spectrum of events and scenes observed under various lighting and weather conditions. In addition, the systems available on the market do not have modules that allow operators to train systems to learn events directly from the video stream. Each function related to adapting the existing video surveillance system involves a tedious process of collecting specific data, developing new models based on it, and then implementing them into the existing infrastructure. Due to the requirements of many algorithms, such implementation often forces companies to replace the existing computing equipment with new ones to support computationally demanding algorithms. For the needs of distributed surveillance systems, the VSaaS service has been introduced, which allows the customer’s attention to focus on specific areas or events that interest them. The provider of such a service bears the equipment costs and maintenance in this variant created on-demand in a distributed environment of many connected cameras or other multimodal devices forming local surveillance systems while simultaneously being part of the global network of the IoT. These local infrastructures are parts of a more extensive ecosystem, causing an even greater demand for algorithms and services to increase their situational awareness of the monitored sites.

The main research directions regarding recognizing people’s actions from a video stream have been presented. These include pose estimation, tracking, deep learning, and space-time-based methods. This part summarizes each of the directions listed above.

### 4.2. Tracking-Based Methods

The current generation of visual trackers has a problem with scene understanding. Existing approaches cannot detect global structures and existent objects or interpret dynamic circumstances meaningfully. In this few-data regime scenario, newer trackers based on adversarial learning may be an alternative and include additional attributes such as spatiotemporal information.

The fundamental goal of modern tracking methods is to create unique neural networks that are simultaneously resilient, accurate, and efficient. Most recent studies have not pre-trained or fine-tuned their backbone networks to utilize generic characteristics and prevent catastrophic forgetting of general patterns. Researchers suggest various machine learning-based strategies to overcome this issue and have demonstrated by preliminary works that adequate backbone network training can improve tracking performance.

Despite significant developments in short-term trackers, long-term trackers are disregarded. On the other hand, long-term trackers seem more useful in real-world circumstances where the tracking object may often disappear or remain occluded for an extended time. After a failure, these trackers should be able to detect the tracking object again and then continue monitoring the proper object in the video stream.

The modern direction—deep learning-based visual tracking approaches have recently examined various uses of deep features, a fusion of hand-crafted and deep features, search strategies, various topologies, and training on datasets, and how to cope with missing training data. However, these are not stable solutions, and many difficulties remain unsolved, and others will need to be investigated further in the future.

Existing tracking-based methods may struggle with tracking multiple people simultaneously, especially when they are close together or appear similarly. Multi-person tracking is a challenging problem in HAR that requires developing effective methods to handle occlusion, appearance changes, and interactions between individuals.

### 4.3. Pose-Based Methods

Despite the promising results, some 2D human pose recognition problems still need to be solved in future studies. Processing efficiency is one of the known problems. Specific frameworks, such as OpenPose, may accomplish near real-time processing on dedicated hardware with a moderate computational capacity. However, more efficient human pose estimation techniques on commercial devices are required in real-world applications.

Another issue is the shortage of data for unusual positions. Whereas existing 2D human pose estimation datasets are big enough for traditional postures, they have inadequate training data for unexpected poses, such as fighting. Model bias and poor performance in unique postures may occur from data imbalance.

The next problem concerns recognizing a person in crowded and natural situations with multiple bodies and other objects occluded. Person detectors may miss the borders of highly overlapping human bodies. In occluded situations, the difficulty of keypoint association is also more evident for bottom-up techniques.

Model generalization is one of the challenges for 3D pose-based methods. Motion capture systems are a bottleneck because they require high-quality 3D ground truth posture annotations, which are expensive and difficult to install in a random environment. As a result, most current datasets have been collected from confined scenarios. On these datasets, state-of-the-art algorithms produce promising results, but their performance declines when applied to real-world data.

The 3D human pose estimation requires substantially more computation than 2D estimation. It is challenging to develop computationally efficient 2D human posture estimate pipelines while keeping high pose estimation accuracy. Due to extreme mutual occlusions and poor resolution content of each individual, the performance of existing 3D human pose estimation algorithms suffer significantly in crowded scenes. Nevertheless, the critical findings are worth discussing since pose-based techniques are one of HAR’s most promising avenues.

### 4.4. Deep Learning-Based Methods

Neural networks are continuously becoming more advanced and are often applied to computer vision problems such as HAR. Some of the modern methods from the above mentioned research also use elements of deep learning—the most studied and utilized methods are CNN and, more recently, ViT. Since ViT is much more advantageous than CNN, we have listed the common disadvantages of these methods that should be considered when selecting these methods.

Transformer models are known for their ability to scale to high levels of parametric complexity, while this is a fantastic trait that enables the formation of massive models, it comes at a hefty cost in training and inference, e.g., according to estimates. The process of training the GPT3 model with 175 billion parameters might cost OpenAI USD 4.6 million. The high computing cost of transformer models also affects computer vision models. Image generators based on sequence-based transformers (such as iGPT) have a high computation cost, limiting their application to high-resolution inputs. In transformers, the time and memory cost of the fundamental self-attention process grows quadratically with the number of image patches.

Transformer designs often require a lot of training to determine the underlying modality-specific principles because they do not natively incorporate prior knowledge to deal with the visual input. The self-attention system must automatically uncover relationships between video frames by analysing an extensive library of video sequences. This process leads to lengthier training durations, higher computational needs, and the processing of big datasets. To achieve a decent performance on the ImageNet benchmark dataset, the ViT [257] model, for example, requires hundreds of millions of pictures. The difficulty of training a transformer in a data-efficient manner is still an open research subject, although recent studies show promising progress.

These significant drawbacks make this direction promising but not mature enough for practical application and research. Nevertheless, the topic of DL to recognize actions in a video is pervasive. There are many comprehensive reviews of the use of classic DL methods to identify human actions [258] as well as video transformers applied to computer vision tasks [233,234,259].

## 5. Conclusions

This paper comprehensively reviews existing human action recognition methods for intelligent video analytics. We examined the advantages and disadvantages of spatio-temporal, pose-based, tracking-based, and deep learning-based approaches, as well as the potential applications of each. Spatio-temporal methods use motion information to capture action patterns, while pose-based techniques utilize body posture to identify human actions. Tracking-based methods use tracking algorithms to identify action sequences, and deep learning-based methods utilize neural networks to classify human activities. Additionally, we compared classic and edge AI intelligent video analytics systems in the cloud, on-premises, and on edge. Popular edge AI neural systems such as Google Coral, Intel Myriad, and Kneros are increasingly used for human action recognition. Google Coral is a system-on-module based on a low-power edge TPU chip, Intel Myriad X is designed for computer vision at the edge, and Kneros is an AI-enabled system-on-module. Deep learning models can be deployed on such neural systems using a wide variety of pre-trained models from the Model Zoo, or custom models can be built and trained with the help of AutoML. Classic AI systems are typically hosted in the cloud, while edge AI systems are designed to run locally on-premises or at the network’s edge. Furthermore, this paper outlined the challenges and opportunities of human action recognition in intelligent video analytics, suggesting possible future research directions. We also provided an in-depth analysis of important aspects of current methods and their potential to improve smart video analytics. Due to the previously mentioned shortcomings of the stability and performance of the presented methods, it is tough to choose one particular class of HAR methods and develop a comprehensive surveillance system enabling the training and real-time recognition of moving objects in broad-spectrum weather conditions. The modern deep learning-based HAR methods show the most promising results but in limited cases. The newest variants of these methods often have high algorithmic and overall computational complexity.

To construct a commercial system to recognize actions from a video stream, each activity in the video data processing pipeline should be explainable, avoiding the unexpected operation of algorithms, and stable, predictable architecture should simplify utilization and allow regular system maintenance and upgrades without the necessity of replacing the current system with an entirely new one.

This review serves as a guide for researchers and practitioners to better understand the last 20 years of research, the current state-of-the-art human action recognition technologies for intelligent video analytics and identify potential opportunities for future research. One classic group of spatio-temporal methods is based on stable, known, and simple spatial and temporal feature detection and description algorithms. Most methods from the spatio-temporal group proved to work fast and predictably with real-world video data in many practical applications. The class of BoVW methods seems to be more robust to environmental changes since they rely on the appearance of objects rather than their spatial relationships. Additionally, BoVW techniques provide better generalization capabilities, as they are less likely to overfit when presented with new data. Finally, BoVW methods are easier to implement and require less training data than other models. Therefore, BoVW methods are good candidates as human action recognition modules in intelligent video analytics before ViT-based methods become more mature with a cheaper entry point. Visual transformers (ViTs) are a more recent approach to image recognition, and have already shown promising results in various computer vision tasks. Unlike BoVW, ViTs rely on self-attention mechanisms to capture the relationships between image features without the need for explicit spatial binning or pooling. This allows ViTs to model more complex and abstract relationships between features, making them more suitable for tasks that require a higher level of understanding of the input images. However, ViTs currently require a large amount of training data and computational resources to achieve a state-of-the-art performance, a major limitation in some applications. In contrast, BoVW methods are relatively simple to implement and require less data to train, making them more suitable for applications with limited data and computational resources.

Overall, both BoVW and ViT approaches have their strengths and weaknesses, and the choice between them depends on the specific requirements and constraints of the application. It is worth remembering that each method has unique characteristics and strengths. A comprehensive approach to analysing human movement may involve combining these methods depending on the specific task.

## Figures and Tables

**Table 1:** Typical workflow elements and operations for implementing IVA systems with HAR methods.

Workflow Element	Operation	HAR Method
Data Acquisition	Capture video data using sensors (e.g., cameras)	-
Pre-processing	Filter, stabilize, and/or enhance the video data	-
Feature Extraction	Extract relevant features from the video data	Pose-Based, Tracking-Based, Spatio-temporal-Based, Deep Learning-Based
Object Detection	Detect objects of interest in the video data	-
Object Tracking	Track objects of interest over time	Tracking-Based
Human Action Recognition	Recognize human actions from video data	Pose-Based, Tracking-Based, Spatio-temporal-Based, Deep Learning-Based
Event Detection	Detect events of interest (e.g., abnormal behaviour)	-
Decision making	Analyse the output of the IVA system and make decisions based on predefined rules	-
Alert Generation	Generate alerts based on the decisions made by the system	-

**Table 2:** Main differences between cloud, hybrid, and edge IVA architectures.

Feature	IVA Architectures		
	Cloud	Hybrid	Edge
System	Centralized	**Decentralized**	**Decentralized**
Power Consumption	**High Compute Power**	**High Compute Power**	Limited Compute Power
Latency	High Latency	**Lowered Latency**	**Lowest Latency**
Bandwidth	High Bandwidth	**Lowered Bandwidth**	**Lowest Bandwidth**
Security	**Secure and Public**	**Secure and Private**	Public
Scalability	**Endless Scalability**	Limited Scalability	Lowest Scalability
Model	**Endless Scalability**	Limited Scalability	Lowest Scalability

**Table 3:** Typical workflow elements and operations for implementing different HAR.

Workflow Element	Pose-Based Methods	Tracking-Based Methods	Spatio-Temporal-Based Methods	Deep Learning-Based Methods
Data Collection	Use sensor devices (e.g., cameras) to capture pose images/videos of humans performing various actions.	Use sensors to capture the movement of the object/person over time.	Use sensors to capture the movement of the object/person over time while also capturing spatial information.	Collect large-scale, labelled datasets of images or videos of humans performing various actions.
Feature Extraction	Extract features from the pose images/videos, such as joint positions, angles, and velocities.	Extract features related to the motion, such as velocity, acceleration, and trajectory.	Extract motion and spatial information features, such as optical flow and histogram of oriented gradients (HOG).	Use deep convolutional neural networks (CNNs) to automatically extract features from raw images or videos.
Action Segmentation	Segment the pose images/videos into individual action sequences.	Segment the object/person movement into individual action sequences.	Segment the object/person movement and spatial information into individual action sequences.	Use recurrent neural networks (RNNs) to segment the video into individual action sequences.
Classification	Classify the individual action sequences into pre-defined action categories.	Classify the individual action sequences into pre-defined action categories.	Classify the individual action sequences into pre-defined action categories.	Use CNNs or RNNs to classify the individual action sequences into pre-defined action categories.
Post-processing	Perform smoothing, filtering, or temporal alignment on the predicted action sequences.	Perform smoothing, filtering, or temporal alignment on the predicted action sequences.	Perform smoothing, filtering, or temporal alignment on the predicted action sequences.	Perform post-processing on the predicted action categories, such as non-maximum suppression or ensembling.

**Table 4:** Workflow of common elements and operations for human action recognition using pose-based methods.

Workflow Elements	Variety of Operations
1. Pose Estimation	(a) Model-based methods, (b) Deep learning methods, (c) Hybrid methods.
2. Feature Extraction:	(a) Handcrafted features, (b) Deep learning-based features.
3. Classification:	(a) SVM, (b) Random Forest, (c) Neural Networks.

**Table 5:** Summary of selected pose estimation methods on various benchmarks.

Method	Accuracy (%)	Joints	Approach	Backbone
OpenPose [106]	93.8	25	Top-Down and Bottom-up	VGG-19
AlphaPose [107]	87.7	18	Top-Down	ResNet
Mask R-CNN [109]	91.4	17	Top-Down	ResNet
HRNet [112]	95.0	17	Bottom-Up	HRNet
DeepCut [113]	91.0	15	Bottom-Up	VGG
DeepPose [114]	70	16	Top-down	ResNet
DensePose [115]	74.7	24	Top-down	ResNet
MediaPipe [116,117]	88.8	33	Bottom-Up	MobileNet
Yolo [118]	-	17	Bottom-Up	CSPDarknet
Kinect SDK [119]	83.5	25	Top-Down	-
wrnchAI [120]	88.4	57	Bottom-Up	-
PoseNet [121]	86.8	17	Top-Down	MobileNet
ST-GCNs [122]	93.2	18	Bottom-Up	-
AGC-LSTM [123]	94.5	25	Top-down	GC-LSTM

**Table 6:** Workflow of common elements and operations for human action recognition using tracking-based methods.

Workflow Elements	Variety of Operations
1. Object Detection	(a) Background subtraction, (b) Haar cascades, (c) Deep learning-based methods.
2. Object Tracking:	(a) Optical Flow, (b) Kalman Filter, (c) Deep learning-based methods.
3. Classification:	(a) SVM, (b) Random forest, (c) Neural Networks.

**Table 7:** Workflow of common elements and operations for human action recognition using spatio-temporal-based methods.

Workflow Elements	Variety of Operations
1. Feature Extraction:	(a) Trajectory-based features, (b) Dense Trajectories.
2. Motion Representation:	(a) Bag of Words, (b) RNNs, (c) CNNs, (d) Transformers.
3. Classification:	(a) SVM, (b) Random forest, (c) Neural Networks.

**Table 8:** Workflow of common elements and operations for human action recognition using deep learning-based methods.

Workflow Elements	Variety of Operations
1. Data Preparation:	(a) Data augmentation, (b) Pre-processing, (c) Data balancing.
2. Network Design:	(a) CNNs, (b) RNNs, (c) Transformers, (d) Attention mechanisms, (e) Hybrid networks.
3. Training:	(a) Backpropagation, (b) Regularization, (c) Optimization.

**Table 9:** Selected datasets for benchmarking human action recognition methods from real-life scenarios.

Name	Videos	Classes
RWF-2000 [183]	2000	2
KTH [246]	2391	6
XD-Violence [247]	4754	9
Weizmann [248]	90	10
UCF sport action [249]	150	10
UCF11 (YouTube) [250]	1160	11
Hollywood2 [179]	1707	12
UCF-Crime [251]	1900	13
Olympic Sports [252]	783	16
UCF50 [253]	6676	50
HMDB51 [177]	6849	51
MultiTHUMOS [254]	400	65
UCF101 [178]	>13,000	101
NTU RGB+D 120 [255]	114,000	120
VMASS2 [180]	>6,000,000	400
Kinetics 700 [256]	650,000	700

## Data Availability

Not applicable.

## Abbreviations

The following abbreviations are used in this manuscript:

IVA	Intelligent video analytics
CCTV	Closed circuit television
CCD	Charge-coupled device
HAR	Human activity recognition
VSaaS	Video surveillance as a service
BoVW	Bag of visual words
RoI	Region of interest
